# A Sanatorium and Market Garden

**Published:** 1906-09-22

**Authors:** Charles Reinhardt

**Affiliations:** Hon. Secretary. The Open-air League, 79 Harley Street, W.


*To the Editor of* THE HOSPITAL.

### A SANATORIUM AND MARKET GARDEN.

*To the Editor of* THE HOSPITAL.

SIR,—The Open-air League, whose general committee includes the Duke and Duchess of Marlborough, Lord and Lady Chelsea, Lord and Lady Iveagh, Lord Monk Bretton, Sir Edward and Lady Sassoon, Sir Edmund Hay Currie, the Hon. W. F. D. and Lady Esther Smith, Mrs. Humphry

Ward, etc., and whose advisory committee includes Drs. James Goodhart, George Heron, Wilfred Hadley, Percy Kidd, and Vaughan Harley, was formed early in the present year to provide inexpensive sanatorium accommodation for the consumptive poor, to educate the public as to the advantages of the open window, and to find occupation for consumptives, cured in sanatoria, which will enable them to escape the dangers which they must incur if they return to the unsuitable conditions under which their health broke down.

The Open-air League is opening its first sanatorium colony at Great Clacton, Essex, in the course of a few weeks. The Institution will be conducted by Dr. John Chapman, who for some years did such valuable work in sanatorium treatment of consumptives, in association with Dr. Noel Bardswell, now medical superintendent of the King's Sanatorium at Midhurst, who is also a member of the committee of the League.

The sanatorium will accommodate twenty-five patients who will be in the incipient stages of the disease, and who will be taught practical market gardening, and who will be kept in residence sufficiently long to enable them to recover their health and to fit them, either independently or in association with the work of the League, to earn a livelihood away from the dangers of town life.

The arrangements that have been made are of so strictly economical a character that the total expenditure will barely exceed fifteen hundred pounds a year, or an average inclusive cost of twenty-five shillings a patient weekly, and it is expected that even this modest figure will be reduced by the value of the work of the inmates.

In the meanwhile the League undertakes a financial responsibility, to enable it to discharge which, it must appeal to the philanthropic public for further funds, and it is hoped that those who recognise the extreme value of the undertaking will respond generously.

Fuller particulars of the objects of the League and of the undertaking herein briefly outlined will be gladly provided on application, and contributions will be gratefully received by the Hon. Treasurer (Lady St. Helier) or by the undersigned.

I am, yours obediently,

Charles Reinhardt.
Hon. Secretary.

The Open-air League, 79 Harley Street, W.,
September 15, 1906.

